# Precision of Classification of Odorant Value by the Power of Olfactory Bulb Oscillations Is Altered by Optogenetic Silencing of Local Adrenergic Innervation

**DOI:** 10.3389/fncel.2018.00048

**Published:** 2018-03-02

**Authors:** Daniel Ramirez-Gordillo, Ming Ma, Diego Restrepo

**Affiliations:** ^1^Department of Cell and Developmental Biology, University of Colorado Anschutz Medical Campus, Aurora, CO, United States; ^2^Rocky Mountain Taste and Smell Center, University of Colorado Anschutz Medical Campus, Aurora, CO, United States; ^3^Neuroscience Program, University of Colorado Anschutz Medical Campus, Aurora, CO, United States

**Keywords:** olfaction, noradrenaline, optogenetics, local field potential, associative learning

## Abstract

Neuromodulators such as noradrenaline appear to play a crucial role in learning and memory. The goal of this study was to determine the role of norepinephrine in representation of odorant identity and value by olfactory bulb oscillations in an olfactory learning task. We wanted to determine whether the different bandwidths of olfactory bulb oscillations encode information involved in associating the odor with the value, and whether norepinephrine is involved in modulating this association. To this end mice expressing halorhodopsin under the dopamine-beta-hydrolase (DBH) promoter received an optetrode implant targeted to the olfactory bulb. Mice learned to differentiate odorants in a go-no-go task. A receiver operating characteristic (ROC) analysis showed that there was development of a broadband differential rewarded vs. unrewarded odorant-induced change in the power of local field potential oscillations as the mice became proficient in discriminating between two odorants. In addition, the change in power reflected the value of the odorant rather than the identity. Furthermore, optogenetic silencing of local noradrenergic axons in the olfactory bulb altered the differential oscillatory power response to the odorants for the theta, beta, and gamma bandwidths.

## Introduction

Axons from noradrenergic (NA) neurons in the locus coreuleus (LC) innervate large areas of the brain where they modulate circuit dynamics (oscillations) in response to changes in behavioral states such as mood, attention and arousal (Bouret and Sara, 2005; Sara and Bouret, 2012; Szabadi, 2013; Aston-Jones and Waterhouse, 2016). While recent studies have confirmed brain-wide innervation by LC-NA neurons (Schwarz et al., 2015; Kim et al., 2016), a recent survey of the divergence of neuronal projections found that LC-NA neurons contain populations with biased output to the olfactory bulb (OB) (Schwarz et al., 2015), raising the question whether selective NA modulation of the OB is involved in sensory processing. Here we characterize local NA neuromodulation of neural oscillatory processing by focal optogenetic silencing of LC-NA axons in a small volume of the OB in a mouse engaged in discriminating between odorants in a go-no go olfactory discrimination task (Li et al., 2015a).

Neuronal oscillations provide a syntactical framework for packaging information into “neuronal letters, words and sentences” for communication between brain areas (Buzsáki, 2010). Local field potential (LFP) oscillations in the olfactory bulb (OB) reflect circuit processing for stimulus detection and sensory decision making for animals learning to differentiate between odorants (Kay, 2014; Frederick et al., 2016). Local infusion of noradrenergic drugs has shown that noradrenergic modulation plays a role in olfactory bulb/piriform cortex circuit processing for successful odorant discrimination by altering mitral/tufted cell synchronized firing, signal to noise ratio in the output to piriform cortex and pattern separation in piriform cortex (Doucette et al., 2007, 2011; Escanilla et al., 2012; de Almeida et al., 2015; Shakhawat et al., 2015). In addition, studies of circuit dynamics in olfactory bulb slices indicate that adrenergic receptor activation leads to long term enhancement of synchronized oscillations in the olfactory bulb (Pandipati et al., 2010), and infusion of the beta noradrenergic blocker propranolol alters the odorant-elicited oscillatory response in the olfactory bulb for the rewarded odorant (Gray et al., 1986).

In this study, we recorded LFP oscillations in the olfactory bulb of mice learning to discriminate between odorants in a self-initiated go-no go odorant discrimination task (Doucette et al., 2011; Li et al., 2015a). Using receiver operating characteristic (ROC) analysis we assessed classification of the identity of the rewarded odorant by the power of the LFP filtered at different bandwidths. We find that as the animal learns to differentiate between odorants there is development of a broadband rewarded odorant-induced change in power of LFP oscillations. Local optogenetic silencing of noradrenergic innervation in the olfactory bulb altered the odorant-induced change in LFP power in the theta, beta, and gamma bandwidths.

## Methods

### Animals

Mice expressing halorhodopsin under the dopamine beta hydrolase promoter (DBH-Cre eNpHR3.0) in LC-NA neurons were produced by crossing DBH-Cre mice (032081-UCD, Mutant Mouse Resource and Research Center) with mice expressing halorhodopsin in a Cre/lox dependent manner [eNpHR3.0, 129S-Gt(ROSA)26Sortm39(CAG-hop/EYFP) Hze/J, Jackson labs] at the University of Colorado Anschutz Medical Campus. DBH is the enzyme that catalyzes the conversion of dopamine to NA and Cre is expressed in LC-NA neurons in DBH-Cre mice (Swanson and Hartman, 1975; Schwarz et al., 2015). For the studies, we used male and female mice 2–5 months of age. Mice were housed in a vivarium with a reversed light cycle of 14/10 h light/dark periods with lights on at 10:00 p.m. Food (Teklad Global Rodent Diet no. 2918; Harlan) was available *ad libitum*. Access to water was restricted to the behavioral session. However, if mice did not obtain ~1 ml of water during the behavioral session, additional water was provided in a dish in the cage (Slotnick and Restrepo, 2005). All mice were weighed daily and received sufficient water during behavioral sessions to maintain >80% of the weight before water restriction. All experiments were performed according to protocols approved by the University of Colorado Anschutz Medical Campus Institutional Animal Care and Use Committee.

### Surgery and optetrode implantation

Male and female mice 2 months of age were anesthetized by brief exposure to isoflurane (2.5%) and subsequently anesthesia was maintained with an intraperitoneal injection of ketamine (100 mg/kg) and xylazine (10 mg/kg). As previously described (Li et al., 2014), the optetrodes included one glass tube for the optic fiber and four tetrodes that consisted of four polyamide-coated nichrome wires (diameter 12.5 μm; Sandvik) gold plated to an impedance of 0.2–0.4 MΩ. Tetrodes were connected and the glass tube was glued through an EIB-16 interface board (Neuralynx). Mice were implanted with an optetrode aimed at the mitral cell layer of the OB 4.25 mm anterior to bregma, 0.4 mm lateral from the midline and 0.53 mm deep measured from the surface of the brain. One ground screw was inserted 1 mm posterior from bregma and 1 mm lateral to the midline and sealed to the bone with dental acrylic. Mice were allowed to recover for 1 week before the initiation of the behavioral studies. All behavioral and LFP recording experiments were performed with mice that had undergone optetrode implantation (2–5 months old). We implanted optetrodes in sixDBH-Cre eNpHR3.0 and eight DBH-Cre mice.

### Go-no go behavioral task

We used the methods previously described in Doucette et al. (2011). Briefly, water-restricted mice were required to enter an odor port and to start licking at the water spout to initiate the release of the odorants 1–1.5 s after port entry. Mice were required to lick at least once in four 0.5 s intervals during reinforced odorant delivery (S+) to obtain 10 μl of water. When exposed to the unreinforced odorant (S–), mice refrain from licking for 2 s because of the effort it takes to lick for this period. Entry of mice into the odor port was detected by breaking the light path of a photodiode, and licking was detected by closing a circuit between the licking spout and the grounded floor of the cage (Slotnick and Restrepo, 2005). The performance of the mice was assessed by calculating correct response to the S+ and S– odorants in 20 trial blocks where 10 S+ and 10 S– odorants were presented at random.

**Figure 1:**
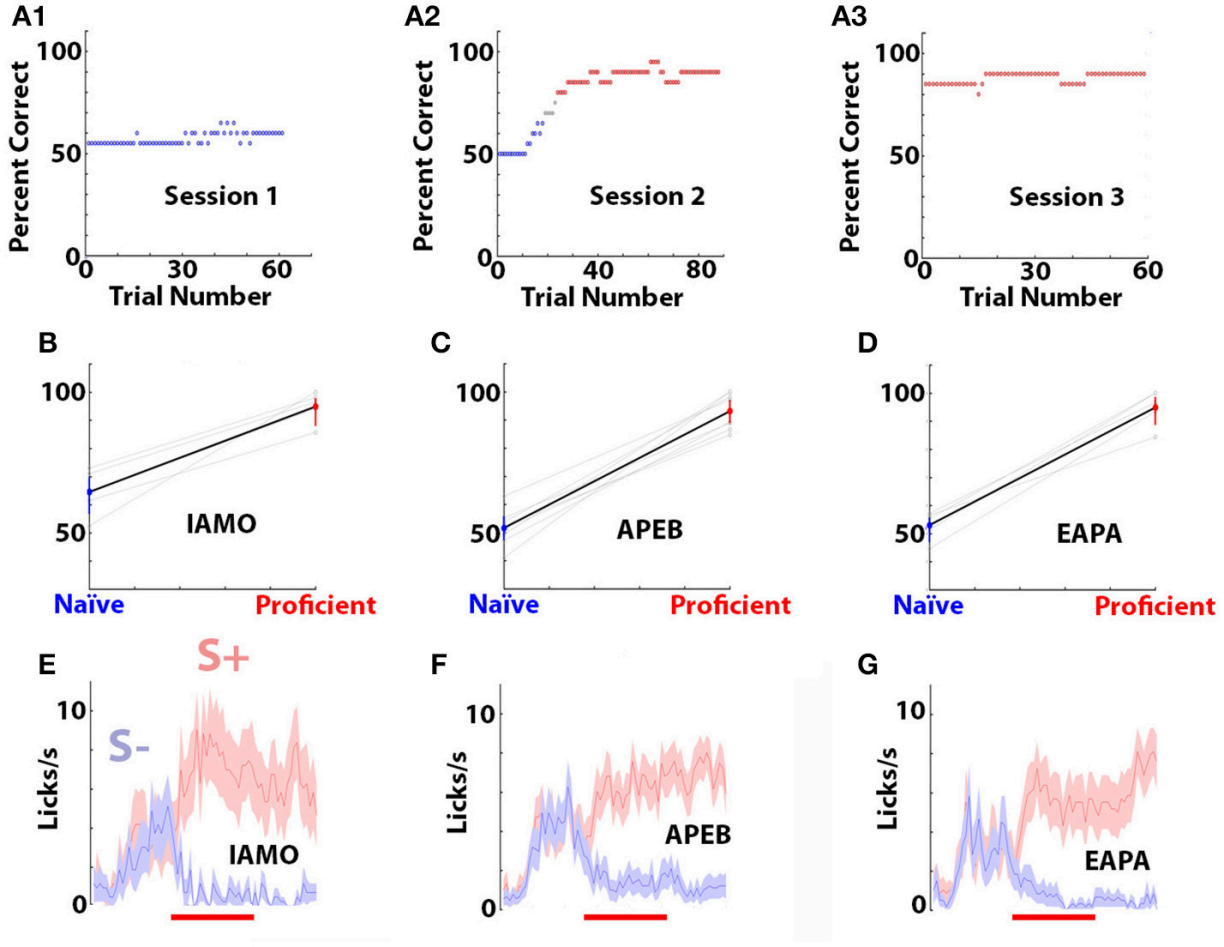
Behavioral performance of the mice in the go-no go task. **(A1–A3)** Example of the percent correct performance in the go-no go odorant discrimination task for a mouse learning to differentiate odorants in the APEB odorant pair. Percent correct was calculated in a window of 20 trials. Blue points are percent correct below 65% and red points are above 80%. **(B–D)** Mean and 95% confidence interval for the percent correct performance in the first thirty trials of the first training session (blue, naïve) and the last thirty trials of the last trainings session (red, proficient). Data for individual mice are shown in light grey. The difference in percent correct performance between naïve and proficient was statistically significant for all odorant pairs (ranksum test, *p* < 0.001, pFDR = 0.05). **(E–G)** Time course of the mean and 95% confidence intervals for the number of licks per second calculated for the last thirty trials of the last session for the same mice. The red line denotes the interval when the odorant was applied for 2.5 s. The odorant pairs and number of mice are: **(B,E)** IAMO, 5, **(C,F)** APEB, 8, **(D,G)** EAPA, 5.

Mice were first trained to discriminate between 1% isoamyl acetate and mineral oil (IAMO odorant pair). Once the mice learned to discriminate between isoamyl acetate and mineral oil the odors were switched to 1% acetophenone (S+, odor) and 1% ethylbenzoate (S–, odor) (APEB odorant pair). Once the mice learned to discriminate between acetophenone and ethylbenzoate the odors were switched to 0.1% ethyl acetate (S+) and 0.05% ethyl acetate + 0.05% propyl acetate (EAPA odorant pair). Mice took 2–6 days to become proficient at discriminating between 1% isoamyl acetate and mineral oil (IAMO), 2–8 days for acetophenone and ethylbenzoate (APEB), and 2–6 days for ethyl acetate vs. ethyl acetate + propyl acetate (EAPA). All odorants were obtained from Sigma-Aldrich and were diluted in mineral oil at room temperature. Mice were trained until they performed at 80 percent correct or better in the last 40 trials in at least two sessions. Figure 1A shows the example of the percent correct odorant discrimination per trial for a mouse that was trained for three sessions in the discrimination of the odorant pair APEB. Percent correct was calculated in a 20 trial window. Figures 1B–D show the mean and 95% confidence interval for the percent correct performance in the first 30 trials of the first session (naïve) and the last 30 trials of the last session (proficient). Figures 1E–G show the time course for the mean number of licks per second and 95% confidence interval for these mice in the last 30 trials of the last session (proficient).

### Optetrode recordings and light stimulation

We followed procedures as previously described (Li et al., 2014). The mouse had access to a chamber with dimensions of 11.6 × 9.7 × 9.4 cm. The EIB-16 board that recorded signals from the tetrodes was connected to either a head stage (LP16CH; Tucker-Davis Technologies) teathered to a 16-channel amplifier (Model 3500, A-M Systems; bandpass, 1–5,000 Hz, 2,000× gain) sampled by a DT3010 analog-to-digital (A/D) card (Data Translation) or it was connected to an INTAN RHD2132 16 channel amplifier/A/D converter that interfaced with an RHD2000 USB interface board. The sampling rate was 24 kHz.

Light was delivered via a diode-pumped, solid-state laser (532 nm; Shanghai Laser and Optics Century) through a 105 μm core diameter 0.22 NA optical fiber (Thorlabs Inc. AFS105/125Y). The power was measured to be 2–10 mW at the end of the optical fiber. Light stimulation was triggered by the computer controlling the olfactometer through a stimulator (Master 8, A.M.P.I.). Light stimulation was presented when the animal entered the port for 3.5 s for both the S– and S+ odorants. The odorant is delivered at a random time 1–1.5 s after the animal enters the odorant port. Optogenetic activation was unilateral. Stimulation through a 0.22 NA optical fiber results in focal light stimulation in a limited volume because of light spread through a cone of 9° whose light intensity is reduced markedly with an exponential-like dependence as a function of depth because of light scatter (Stujenske et al., 2015). Limited unilateral optogenetic activation of eNpHR3.0 was performed on purpose to study changes in circuit processing due to silencing of local noradrenergic innervation. Local unilateral silencing is not expected to result in substantial changes in behavior.

### LFP analysis

LFP recordings from each of the 16 electrodes were analyzed by computing the mean spectral power computed within a sliding 1 s window using the MATLAB spectrogram function. The power, computed in decibels, was shown either as a time course for the mean power for a subset of trials, or calculated as the odorant-induced change in power (Δ power). Δ power was calculated as the average power, computed in decibels, for 2 s during odorant application minus the average power in the interval from 2.1 to 0.6 s before exposure to the odorant. The bandwidths used to filter the oscillations were defined as theta (6–12 Hz), beta (15–30 Hz), low gamma (35–55 Hz), and high gamma (65–95 Hz). Receiver operating characteristic analysis (ROC) was used to assess the classification of the rewarded and unrewarded stimuli using Δ power (Fawcett, 2006). ROC was estimated using the roc function from MATLAB exchange (Cardillo, 2008). The area under the ROC curve was defined from −0.5 to 0.5 with a value of zero when the ROC fell on the diagonal. The significance of the auROC was estimated calculating the *p*-value using a *z*-test (Cardillo, 2008). The significance of the differences in auROC calculated from Δ power values for the LFP recorded by each electrode was estimated using a non-parametric permutation based ANOVA (Delorme and Makeig, 2004). The *p*-value for significance was estimated for multiple comparisons using the false discovery rate (Curran-Everett, 2000). The MATLAB code used to process the data is available at https://github.com/restrepd.

### Analysis of lick-aligned LFP

We used the methods of Amarante et al. (2017). Briefly, we aligned the raw LFP to the onset of the licks, and we used spectrogram analysis of LFP with a sliding 1 s window to determine the dependence of the LFP power in the different frequencies as a function of the time to the onset of the licks. In addition, we used the Hilbert transform to determine the theta LFP phase of the licks.

### Histochemical characterization of EYFP co-expressed with halorhodopsin in eNpHR3.0

Mice aged two months old were perfused with cold phosphate-buffered saline and then 4% paraformaldehyde in PBS. The brain was post-fixed in 4% paraformaldehyde overnight at 4°C and cryoprotected with 30% sucrose. Coronal sections (60 μm thick) were cut with a freezing cryostat and then mounted with Vectashield media (H-1500, Vector Laboratories). Fluorescently labeled cells were detected on a confocal microscope (Leica SP5) using a 10× objective and images were later processed with ImageJ.

## Results

The goal of this study was to determine whether local adrenergic innervation modulates LFP oscillations in the olfactory bulb in awake behaving mice discriminating odorants in the go-no go associative learning task. For three different odorant pairs we determined whether odorant-induced changes in oscillatory power differed between the rewarded and unrewarded odorants, and whether this odorant-induced change in oscillatory power changes as the animal learns to differentiate between the odorants. Finally, we asked whether *local* optogenetic silencing of the adrenergic fibers in the OB of DBH-Cre transgenic mice expressing halorhodopsin (eNpHR3.0) alters the odorant-elicited change in oscillatory power.

For mice proficient in discrimination of odorant pairs the rewarded odorant elicits a broadband increase in oscillatory power, and ROC analysis indicates that the odorant-induced change in LFP power can be used to classify the two odorants as rewarded vs. unrewarded.

**Figure 2:**
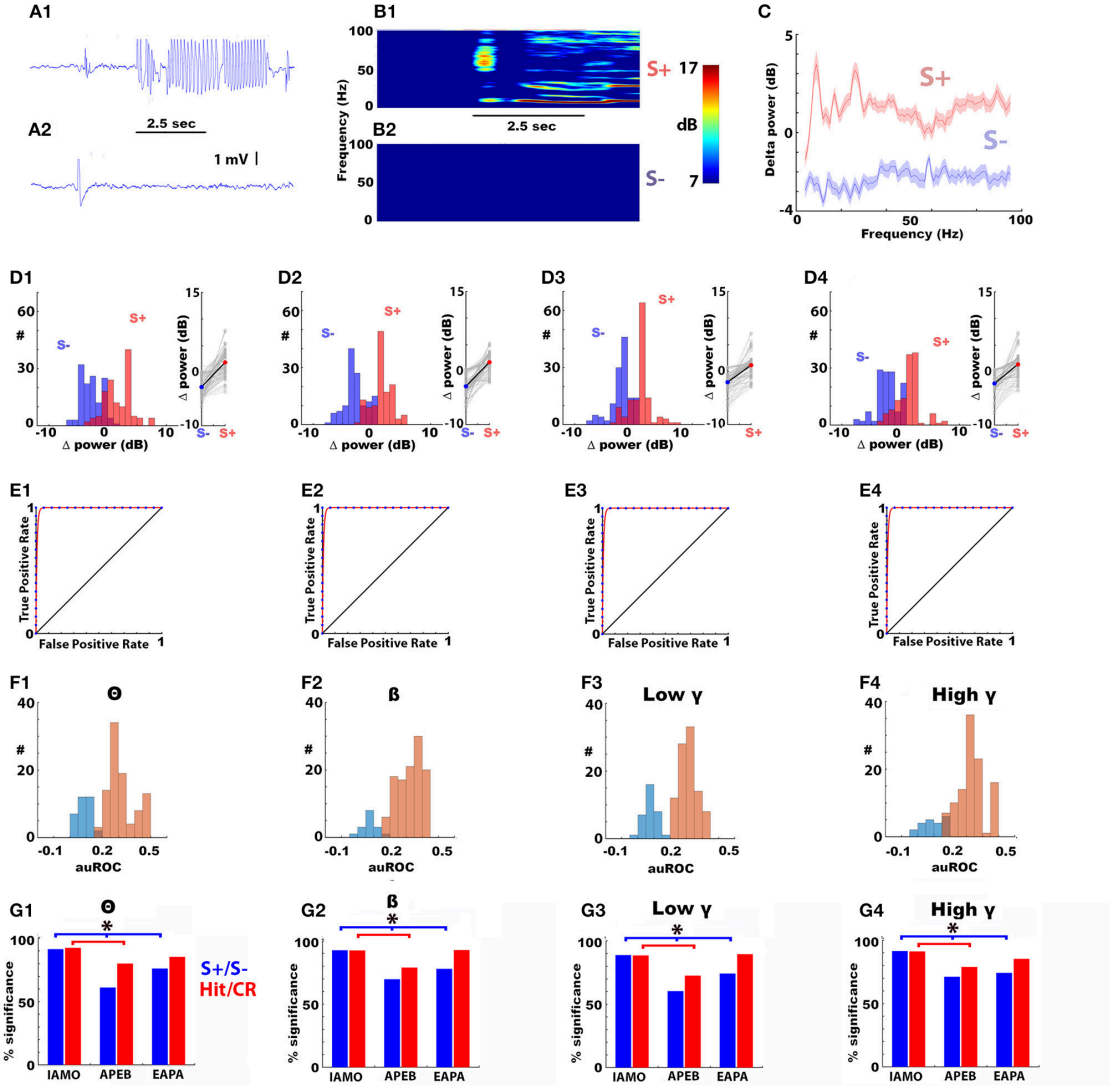
The rewarded (S+) and unrewarded (S–) odorants elicit broadband increases and decreases in LFP power in the olfactory bulb in mice proficient in the go-no go odorant discrimination task. Panels **(A1,A2)** show representative examples of the raw traces of the olfactory bulb LFP in response to the S+ (**A1**, top) and S– (**A2**, bottom) odorants for the APEB odorant pair. The mouse was exposed to the odorant for 2.5 s (black bar). In A to F the S+ odorant was 1% acetophenone diluted in mineral oil (AC) and the S**–** stimulus was 1% ethylbenzoate (EB) (APEB odorant pair). Panels **(B1,B2)** shows an example of the time course for the average Δ power spectrogram (in decibels) for the response to the S+ (**B1**, top) and S– (**B2**, bottom) odorants for the last 30 trials in the last training session where this animal reached >80% percent correct responses (“proficient”). Δ power used for panels **(C–G)** was calculated as the average power during the first 2 s of odorant application, computed in a logarithmic scale, in a sliding 1 s window during odorant application minus the average power in the interval from 2.1 to 0.6 s before exposure to the odorant. Panel **(C)** shows the average Δ power spectrum calculated during odorant application for frequencies spanning theta to high gamma calculated from LFP measurements in 16 electrodes in the last 30 trials of the last training session for six mice. The shadow displays a 95% confidence interval. Panels **(D1–D4)** shows for four different bandwidths ranging from theta to high gamma the histogram for the number of LFP recordings per electrode at each average Δ power and scatter plots for the average Δ power calculated in the last 30 trials for each LFP recorded from each electrode in the six mice discriminating odorants in the APEB odorant pair. In the plot on the right side of the histogram the solid line shows the average Δ power. Panels **(E1–E4)** shows examples of the receiver operating characteristic (ROC) graphs (Fawcett, 2006) estimated from the S+ and S– Δ power distributions for the last 30 trials of the last go-no go session for training a mouse to differentiate between AC (S+) and EB (S–). The blue points are the true positive and false positive rates for each trial and the red line is a best fit of the ROC curve. The area under the ROC (auROC, defined here to fall between −0.5 and 0.5) was significantly different from zero (the diagonal) using a *z*-test (Cardillo, 2008) (*p* < 0.05). Panels **(F1–F4)** shows the histogram of the auROCs calculated for the last 30 trials in the last go-no go training session for each electrode LFP in each bandwidth for all electrodes recorded from in the six mice (all S+ and S– trials were included in the ROC calculation). Significant auROCs are shown in light brown, and auROCs that were not statistically significant are shown in light blue. auROC significance was tested using the *z*-test and the *p*-values were corrected for multiple comparisons by calculating the significance *p*-value corrected for the false discovery rate (Curran-Everett, 2000) (pFDR = 0.04). Panels **(G1–G4)** show the percent of single electrode LFP auROCs significantly different from zero for the different odorant pairs used in this study. The ROCs for the blue bars were calculated using all S+ and S– trials, the ROCs for the red bars were calculated using only Hit and CR trials. S+/S– odorants: IAMO: 1% isoamyl acetate/mineral oil, APEB: 1% acetophenone/1% ethylbenzoate and EAPA: 0.1% ethyl acetate/0.05% ethyl acetate + 0.05% propyl acetate. ^*^The *p*-value for a Chi-Squared testing for the difference in the number of significant LFPs is smaller than the pFDR = 0.037). Number of mice: IAMO: 5, APEB: 8, EAPA: 5, 16 electrodes per mouse). The behavioral performance is shown in Figure 1.

We recorded the LFP in the OB of mice proficient (>80 percent correct) in discriminating two odorants in the go-no go operant conditioning task where thirsty mice receive a water reward if they lick for 2 s when presented with the rewarded odorant (S+), but do not obtain the reward for the unrewarded odorant (S–) regardless of licking. Figures 2A1,A2 show the raw LFP trace for a trial where the mouse responded to the rewarded odorant (1% acetophenone, Figure 2A1) vs. the unrewarded odorant (1% ethylbenzoate, Figure 2A2, APEB odorant pair). A spectrogram analysis of the change in power elicited by the odorant (Δ power) in this experiment shows a broadband increase in power for frequencies between 4 and 100 Hz for the rewarded odorant, but not for the unrewarded odorant for the last 30 trials in the last training session where the animal reached >80% percent correct responses (Figures 2B1,B2). Figure 2C shows the spectrogram for the average and 95% confidence interval for the Δ power calculated for 30 trials from all electrode LFP recordings for a window of 2 s after addition of the odorant from 6 mice proficient in discrimination of 1% acetophenone and 1% ethylbenzoate (APEB odorant pair). Figures 2D1–D4 show histograms and point plots for the LFP Δ power calculated from each electrode in these 6 mice for the rewarded and unrewarded odorants. The S+ trials show larger LFP Δ power than the S– trials suggesting that Δ power can differentiate between the two odorants.

Whether Δ power can be used to differentiate between the two odorants was evaluated with ROC analysis (Figures 2E–G). Figure 2E shows the ROC calculated with S+ and S– trials for the recordings whose spectrogram is shown in Figure 2B. the area under the ROC (auROC) was significantly different from zero for all bandwidths (zero is an ROC curve falling at the diagonal, auROC ranges from 0.5 to −0.5). Figure 2F shows histograms of the auROCs for all recordings from the six mice discriminating the APEB odorant pair calculated using S+ and S– in the last 30 trials. The significant auROCs are shown in brown, and the auROCs that were not significant are shown in blue. auROC significance was tested using the *z*-test and the *p*-values were corrected for multiple comparisons by calculating the significance *p*-value corrected for the false discovery rate (Curran-Everett, 2000) (pFDR = 0.046). The majority of auROCs were significant (Figure 2G).

The auROC analysis calculated using S+ and S– trials was repeated for all mice with two other odorant pairs. One odorant pair was 1% isoamyl acetate vs. mineral oil (IAMO odorant pair) while the other odorant pair was a chemical vs. a mixture of two chemicals: 0.1% ethyl acetate/0.05% ethyl acetate +0.05% propyl acetate (EAPA odorant pair). Most of the recordings yielded significant S+/ S– auROCs for Δ power with the two different odorants and the percent significant auROCs was smaller for the APEB and EAPA odorant pairs compared to the IAMO odorant pair for all bandwidths (Figures 2G1–G4, blue bars, the *p* < pFDR = 0.019 for a Chi-Squared testing for the difference in the number of significant LFPs, number of mice: IAMO: 5, APEB: 8, EAPA: 5, 16 electrodes per mouse). Finally, we performed the ROC analysis using only Hit an CR trials found in the last 30 trials. The red bars in Figures 2G1–G4 show that a substantial percent of these Hit/CR auROCs were significant.

### The difference between the rewarded and unrewarded odorants in the odorant-induced change in oscillatory power becomes larger when the animal becomes proficient in differentiating between the odorants

We examined whether the difference between the rewarded and unrewarded odorants in the odorant-induced change in broadband oscillatory power (Δ power) developed as the animal learned to differentiate between odorants. We compared the auROC of rewarded (S+) vs. unrewarded (S–) odorant Δ power in the first 30 trials of the first go-no go learning session (naïve) with the last 30 trials of the session where the animal had become proficient. The percent correct performance in the first 30 trials of the first go-no go training session (naïve) and the last 30 trials of the last training session (proficient) for the different odorant pairs and the time course for licking during the trial in proficient mice are shown in Figure 1.

**Figure 3:**
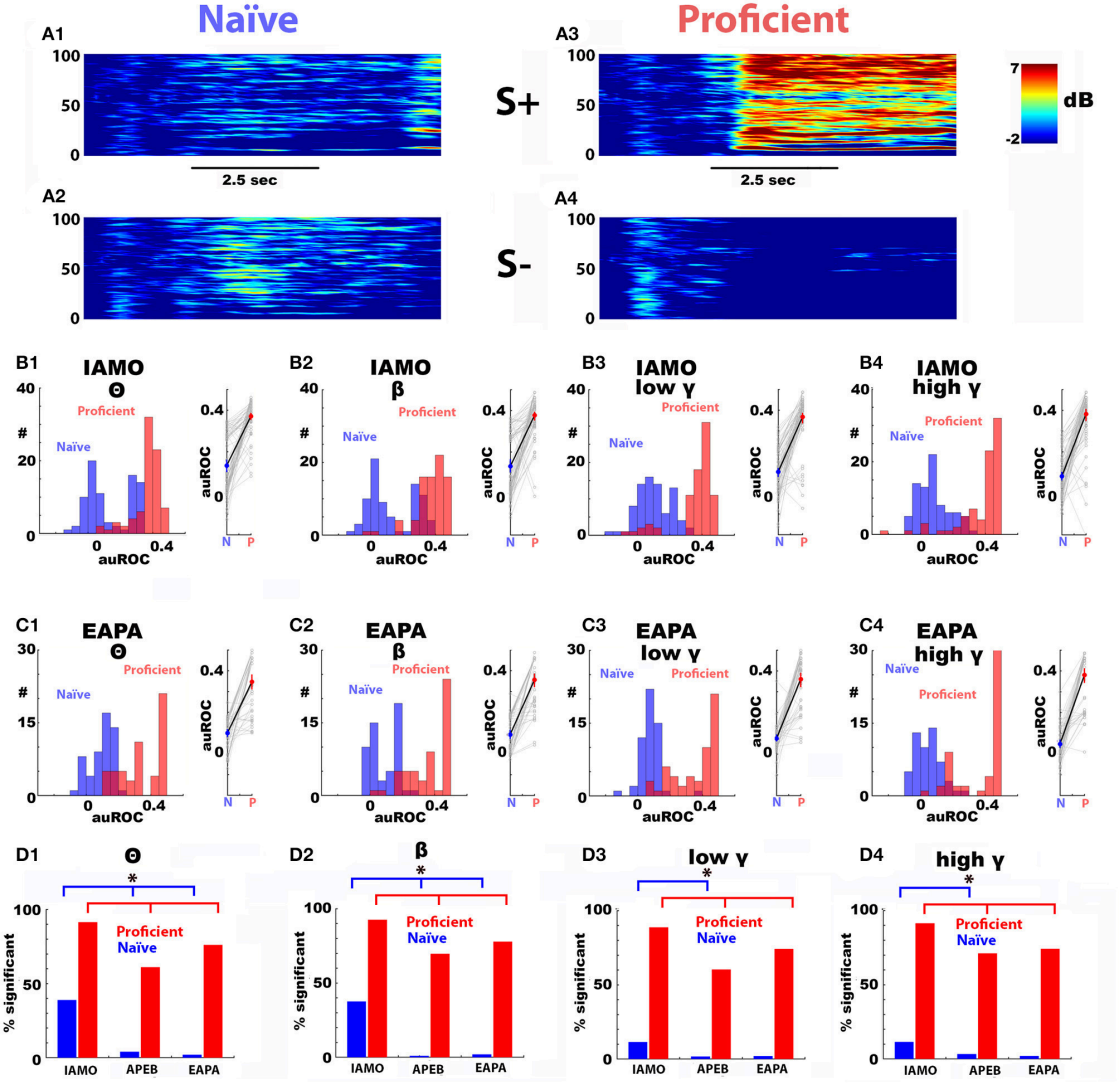
The difference between the rewarded and unrewarded odorant-elicited change in LFP power develops as the mice become proficient in learning to discriminate between the odorants. Panels **(A1–A4)** show an example of the increase in the odorant-induced change in power (Δ power) elicited by the reinforced odorant as a mouse became proficient in discriminating between the rewarded (IA: 1% isoamyl acetate) and unrewarded (mineral oil: MO) odorants. Panels **(A1,A2)** are the spectrograms for the average Δ power in decibels for first 30 trials in the first go-no go session where mice learned to discriminate between IA (S+, **A1**) and MO (S–, MO)(naïve period). Panels **(A3,A4)** are the average Δ power spectrograms for the last 30 trials in the last session for S+ **(A3)** and MO **(A4)** (proficient period). Black bar: duration of odorant application. Panels **(B1–B4)** are histograms (left) and scatter plots (right) for the auROCs for the average Δ power elicited by S+ vs. the average Δ power elicited by S– for the different bandwidths for the naïve period for IA vs. MO (blue) and proficient period (red) for LFPs measured in 16 electrodes in five mice. The difference in Δ power auROCs between learning and proficient is statistically significant for all bandwidths when tested using a non-parametric permutation based ANOVA (Delorme and Makeig, 2004) (*p* < 0.001, pFDR = 0.05). Panels **(C1–C4)** are auROCs for the average Δ power during naïve and proficient periods for the different bandwidths for 0.1% ethyl acetate/0.05% ethyl acetate + 0.05% propyl acetate (EAPA odorant pair). The difference in Δ power auROCs between naïve (blue) and proficient (red) is statistically significant for all bandwidths when tested using a non-parametric permutation based ANOVA (Delorme and Makeig, 2004) (*p* < 0.001, pFDR = 0.05). Panels **(D1–D4)** show the percent of single electrode LFP auROCs significantly different from zero for the different odorant pairs used in this study. S+/S– odorants: IAMO: 1% isoamyl acetate/mineral oil, APEB: 1% acetophenone/1% ethylbenzoate and EAPA: 0.1% ethyl acetate/0.05% ethyl acetate + 0.05% propyl acetate. ^*^The *p*-value for a Chi-Squared testing for the difference in the number of significant LFPs is smaller than the pFDR (pFDR = 0.037). Number of mice: IAMO: 5, APEB: 8, EAPA: 5, 16 electrodes per mouse. The auROC histograms are not shown for APEB. The behavioral performance is shown in Figure 1.

Figure 3A shows an example of the time course for the spectrogram for Δ power during the first thirty trials when the animal was naïve to the value of the odorants (Figures 3A1,A2) and for the last 30 trials when the animal was proficient in discriminating 1% isoamyl acetate from mineral oil (IAPA odorant pair) (Figures 3A3,A4). We observed a marked broadband increase in rewarded (S+) odorant-induced Δ power. Figures 3B,C show histograms and scatter plots for all auROCs computed for Δ power elicited by the IAMO and EAPA odorant pairs during the naïve and proficient periods in the go-no go sessions (number of mice: IAMO: 5, EAPA: 5, 16 electrodes per mouse). For both odorant pairs, there was a significant increase in the auROC as the animals became proficient in discriminating the odorants. The difference in Δ power auROCs between naïve (blue) and proficient (red) is statistically significant for both odorant pairs for all bandwidths when tested using a non-parametric permutation based ANOVA (Delorme and Makeig, 2004) (*p* < 0.001, pFDR = 0.05).

Interestingly, for the IAMO odorant pair where the animals perform discrimination of isoamyl acetate vs. mineral oil, a subset of auROCs were significant in the first 30 trials of the first training session (naïve, Figures 3D1–D4). In contrast, for discrimination of an odorant from an odorant mixture (EAPA: 0.1% ethyl acetate/0.05% ethyl acetate + 0.05% propyl acetate) there were a smaller number of significant auROCs in the naïve period (Figures 3D1–D4). On the other hand, when the animals became proficient, the percent of significant auROCs was smaller for the EAPA and APEB odorant pairs than the IAMO pair for all bandwidths (Figures 3D1–D4). The statistical difference in the percent of significant auROCs was assessed with a Chi-Squared test (*p* < pFDR, pFDR naïve = 0.044, pFDR proficient = 0.019). Finally, the broadband increase in Δ power as the animal learned to discriminate the odorants was not due to a steady change in electrode impedance because when the animals were exposed to a new odorant pair the percent of significant auROCs decreased markedly for the first 30 trials (the order of odorant pair go-no go sessions was: IAMO, APEB, and EAPA).

### The odorant-induced change in LFP power differs between false alarm and correct rejection

**Figure 4:**
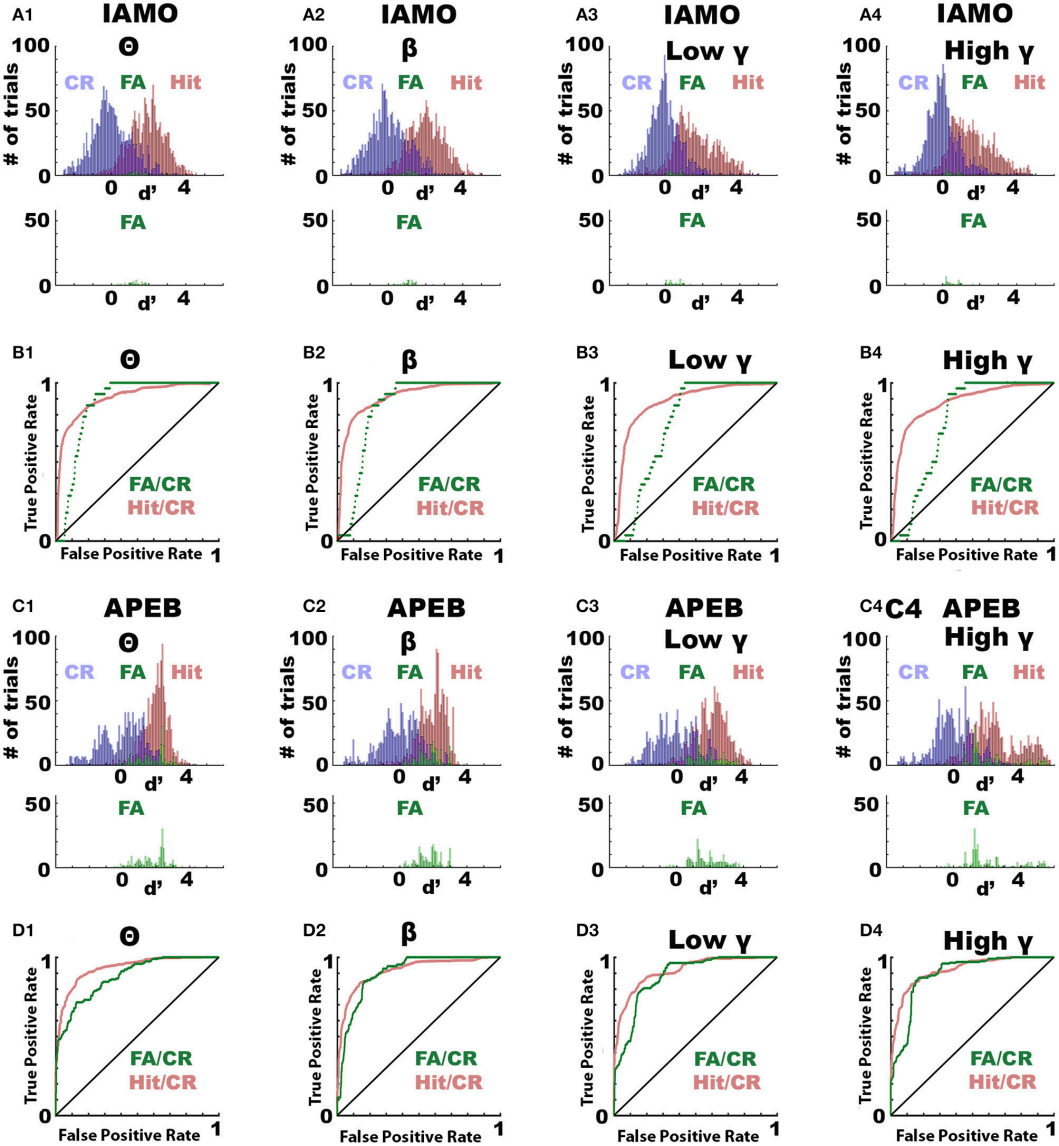
ROC analysis of the odorant-elicited change in LFP power (Δ power) for trials when the animal responds by licking to the unrewarded odorant (false alarm, FA). ROC analysis of the per trial LFP Δ power calculated when the animals were performing >80% correct. ROC was calculated for Δ power calculated in FA trials vs. Δ power calculated in correct rejection (CR) trials where the animal refrained for licking to the unrewarded odorant (FA/CR) or for Δ power calculated in Hit trials where the animal was licking for the rewarded odorant vs. Δ power calculated in CR (Hit/CR). **(A,B)** IAMO odorant pair, **(C,D)** EAPA odorant pair. **(A,C)** Histograms for LFP Δ power in decibels for Hits (red), CRs (blue) or FAs (green). **(B,D)** ROC analysis for Hit/CR (red) or FA/CR (green). All auROCs were significantly different from the diagonal (*p* < 0.0001, pFDR = 0.05). auROC significance was tested using the *z*-test and the *p*-values were corrected for multiple comparisons by calculating the significance *p*-value corrected for the false discovery rate (Curran-Everett, 2000).

Next, we asked the question whether the odorant-induced change in LFP power (Δ power) differed between trials when the animal licked for the unrewarded odorant (false alarms, FA) compared to trials when the animal refrained from licking (correct rejections, CR). We performed this analysis by computing ROC for all trials for the different odorant pairs for animals that were proficient (percent correct >80%). Δ power was normalized (*d*′) by dividing by the average standard deviation of the Δ power distributions. Figures 4A1–A4,C1–C4 show the *d*′ distributions for Hit, CR and FA for the IAMO and APEB odorant pairs in all frequency bandwidths. As shown, *d*′ for FA was larger than for CR suggesting that Δ power differs when the animal makes a mistake in responding to unrewarded odorant. Figures 4B1–B4,D1–D4 show a ROC analysis for *d*′. The auROC was significantly different from zero (the diagonal) for all frequency bandwidths for both the FA/CR and Hit/CR *d*′ distributions (*p* < pFDR = 0.05). Thus, odorant-induced Δ power at all bandwidths performs relatively well in classifying correct from incorrect responses suggesting that Δ power reflects odorant value as opposed to odorant identity.

### The odorant-induced change in LFP power changes polarity when the reward is reversed

**Figure 5:**
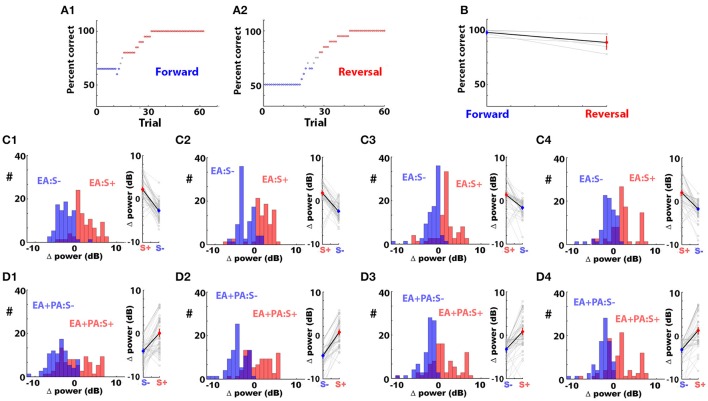
The odorant-elicited change in LFP power (Δ power) reverses polarity when the rewarded odorant is reversed. **(A1)** Percent correct discrimination as a function of trial number for a mouse becoming proficient in differentiating 0.1% ethyl acetate (EA) as the rewarded odorant from a mixture of 0.05% ethyl acetate and 0.05% propyl acetate (EA+PA) as the unrewarded odorant (forward session). **(A2)** Percent correct discrimination as a function of trial number for the same mouse learning to differentiate these two odorants after the reward was shifted to EA+PA (reversed session). Percent correct was computed in a window of 20 trials. The points are blue when percent correct <65% and red when percent correct >80%. **(B)** Percent correct for the last 30 trials for the forward and reversed go-no go sessions for five different mice. A ranksum test indicates that the percent correct was different between the forward and reversed sessions (*p* = 0.04). **(C,D)** Histograms and point plots of the mean LFP Δ power computed in the last 30 trials of the forward and reversed go-no go sessions for EA **(C)** and EA+PA **(D)** in all bandwidths (LFP was recorded from 16 electrodes in 5 mice). A permuted ANOVA test of the difference in LFP Δ power between the forward and reversed sessions yielded a *p* < 0.0001 for all bandwidths and for both odorants (pFDR = 0.05).

These results raised the question whether reversal of the reward would change the polarity of the odorant-induced change in LFP power. We performed experiments where the animal became proficient at differentiating 0.1% ethyl acetate (EA) as the rewarded odorant from a mixture of 0.05% ethyl acetate and 0.05% propyl acetate (EA+PA) as the unrewarded odorant in the go-no go task (EAPA odorant pair, forward go-no go task). Once the animal became proficient, we reversed the reward making EA+PA the rewarded stimulus. Figures 5A,B show that the animals were able to differentiate between the odorants in both conditions by responding to the rewarded odorant. We found that the odorant-induced change in power of the LPF oscillations reversed polarity at all bandwidths when the rewarded stimulus was reversed (Figures 5C,D). The *p*-value for the permuted ANOVA testing for the difference in Δ power between the rewarded and unrewarded odorants was smaller than 0.0001 for all the bandwidths for both forward and reverse sessions (Pfdr = 0.05).

### Local optogenetic silencing of noradrenergic axons in the olfactory bulb does not elicit changes in the area under the ROC for the odorant-induced change in LFP power evaluated with hit and correct rejection trials

**Figure 6:**
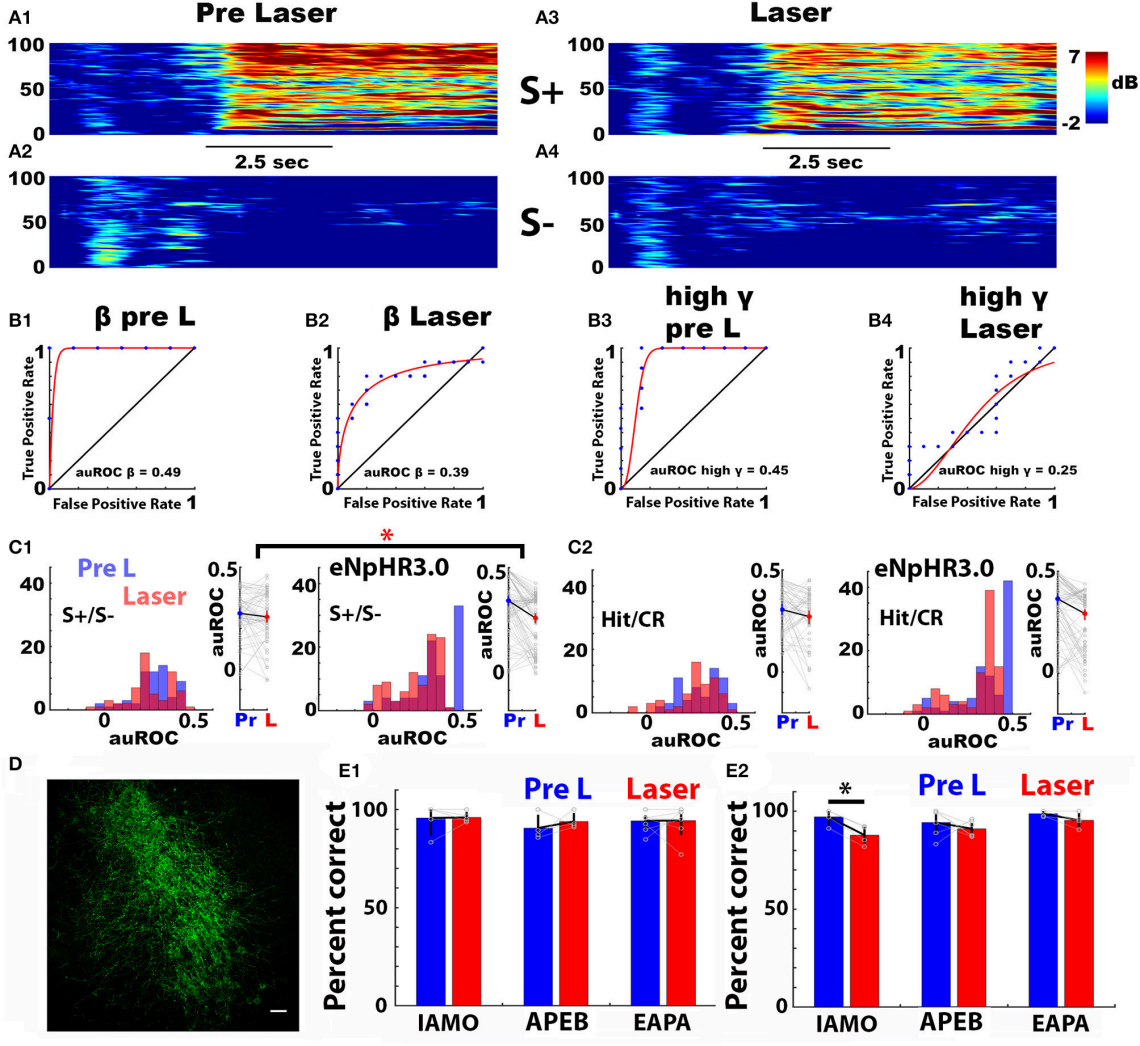
Optogenetic silencing of norepinephrine axons in the olfactory bulb does not alter the broadband odorant-elicited change in LFP power (Δ power). **(A1–A4)** Example of the Δ power spectrograms recorded in one go-no go session with a DBH-Cre eNpHR3.0 mouse for S+ (0.1% ethyl acetate, **A1, A3**) and S– (0.05% ethyl acetate + 0.05% propyl acetate, **A2,A4**) for 20 trials before **(A1,A2)** and 20 trials during **(A3,A4)** local optogenetic activation in the OB of eNpHR3.0 expressed in noradrenergic axons. The laser was activated for 3.5 s starting at the time the mouse entered the odorant port, 1–1.5 s before odorant application. Black bar: Duration of odorant stimulation. **(B1–B4)** Examples of auROCs for Δ power for S+ (0.1% ethyl acetate) and S– (0.05% ethyl acetate + 0.05% propyl acetate) (EAPA odorant pair) for 20 trials before **(B1,B3)** and 20 trials during **(B2,B4)** local optogenetic activation of eNpHR3.0 expressed in noradrenergic axons in the OB. **(B1)** auROC for beta LFP Δ power before laser stimulation, **(B2)** auROC for beta LFP Δ power during laser stimulation. **(B3)** auROC for high gamma LFP Δ power before laser stimulation and **(B4)** auROC for high gamma LFP Δ power after during laser stimulation. **(C1,C2)** Histograms (left) and scatter plots (right) for Δ power auROCs for theta bandwidth before (Pre L, blue) and during (Laser, red) laser stimulation for the APEB odorant pair. **(C1,C2, left)** DBH-Cre mice (*n* = 4, 16 LFP electrodes each), **(C1,C2, right)** DBH-Cre eNpHR3.0 mice (*n* = 6, 16 LFP electrodes each). The auROC in **(C1)** was calculated using LFP Δ power recorded in S+ vs. S– trials. The auROC in **(C2)** was calculated using LFP Δ power recorded in Hit vs. CR trials. The interaction term of an N-way ANOVA with mouse genotype and laser stimulation as the two factors was significant for the S+/S– auROC (**C1**) (*p* = 0.016 < pFDR = 0.031), but was not significant for the Hit/CR auROC (**C2**) (*p* = 0.19 >pFDR = 0.031). **(D)** Expression of EYFP in the locus coreuleus of DBH-Cre eNpHR3.0 mice. **(E)** Performance of mice in the go-no go odorant discrimination task for the last 20 trials in the last training session (blue, Pre L), and in the first 20 trials in the subsequent training session where the laser was turned on for the duration of odorant delivery (red, Laser). Results are shown for the three odorant pairs for DBH-Cre mice **(E1)**, and DBH-Cre eNpHR3.0 mice **(E2)**. A paired *t*-test yields a significant difference in performance between the two sessions for DBH-Cre eNpHR3.0 mice discriminating between the IAMO odorants (*p* = 0.002, pFDR = 0.008, *n* = 4). The number of DBH-Cre mice tested was 5 for IAMO, 4 for APEB, and 8 for EAPA, and the number of DBH-Cre eNpHR3.0 mice was 4 for IAMO. Six for APEB and 4 for EAPA.

Next, we asked whether local optogenetic silencing of noradrenergic axons in the OB altered auROC for the odorant-induced LFP Δ power. We silenced LC axons in the OB through unilateral focal light stimulation of the inhibitory opsin eNpHR3.0 in the vicinity of the tetrodes in DBH-Cre eNpHR3.0 mice that express the opsin in noradrenergic neurons. The laser was turned on for 3.5 s starting when the animal entered the odor port. DBH-Cre eNpHR3.0 mice expressed eNpHR3.0 in LC (Figure 6D). As a control, we used DBH-Cre mice that do not express eNpHR3.0. The analysis was performed for the last 20 trials of the last session when mice became proficient at differentiating the odorant pairs (pre-laser, or Pre L), and for the first 20 trials of the subsequent session when the laser was turned on when the animal entered the odorant port (Laser).

Figure 6A shows examples of the time course of the spectrogram of the odorant-induced LFP Δ power elicited by the rewarded odorant in a DBH-Cre eNpHR3.0 mouse for the EAPA odorant pair for the 20 trials before (Figures 6A1,A2) and during (Figures 6A3,A4) opsin activation and Figures 6B1–B4 show two examples of a light-induced decrease the auROC calculated using S+ and S– trials for beta and high gamma for the EAPA odorant pair. We proceeded to perform a ROC analysis for the difference in light-induced silencing of NA fibers in mice expressing, or not expressing eNpHR3.0 (DBH-Cre eNpHR3.0 vs. DBH-Cre mice). Figure 4C1 shows histograms and scatter plots of the effect of local optogenetic silencing on the LFP Δ power auROC calculated for S+ and S– for 20 trials for the theta bandwidth for control DBH-Cre (Figure 6C1, left) and DBH-Cre eNpHR3.0 (Figure 6C1, right) mice discriminating the APEB odorant pair. auROC values are shown for the last 20 trials of the session when the mice had become proficient at odorant discrimination (Pre L, blue) and for the first 20 trials for the subsequent session when the laser was turned on when the animal entered the odorant port (Laser, red). The auROC appears to decrease when the noradrenergic fibers are silenced by optogenetic activation of eNpHR3.0 (Figure 6C1, right). An N-way ANOVA for the interaction between light activation and genotype for the Δ power auROC was significant for the data in Figure 6C1 (APEB odorant pair, theta bandwidth, *p* = 0.02 < pFDR = 0.031, 6 DBH-Cre eNpHR3.0 mice and 4 DBH-Cre, 16 electrodes each). The N-way ANOVA also found that there was a differential effect of silencing NA fibers for the two genotypes for the high gamma bandwidth of the Δ power auROC calculated for S+ and S– for mice discriminating the IAMO odorant pair (data not shown, *p* = 0.03 < pFDR = 0.05, for IAMO we used 4 DBH-Cre eNpHR3.0 mice and 6 DBH-Cre mice, 16 electrodes each). However, there was not a statistically significant differential effect of silencing NA fibers for the two genotypes for the Δ power auROC calculated for S+ and S– for any of the other bandwidths for IAMO and APEB, or any of the bandwidths of EAPA (*p* > pFDR, pFDR for EAPA was 0.05, 4 DBH-Cre eNpHR3.0 mice and 8 DBH-Cre, 16 electrodes each).

A question that arises is whether the differential effect of optogenetic NA fiber silencing for the two genotypes for the auROC calculated for S+ and S– is due to a change in the behavioral performance of the mice. Indeed, Figure 6E2 shows that optogenetic silencing of noradrenergic fibers elicits a significant decrease in the performance of the DBH-Cre eNpHR3.0 mice engaged in discriminating the IAMO odorant pair (paired *t*-test *p* = 0.0017 < pFDR = 0.008). In order to determine whether the effects of optogenetic silencing on the auROC was due to changes in behavioral performance we re-calculated the auROC using only Hits and CRs. When the Δ power auROC was calculated for Hits and CRs the N-way ANOVA did not find significant genome x light interaction effects of optogenetic silencing of NA fibers in the OB for any of the odorant pairs and LFP bandwidths (*p* > pFDR, pFDR = 0.05 for IAMO, 0.03 for APEB and 0.006 for EAPA, an example of the Hit/CR auROC is shown in Figure 6C2). This suggests that the effect of optogenetic silencing of noradrenergic fibers on the auROC is due to the change in behavioral performance.

### Local optogenetic silencing of noradrenergic axons in the olfactory bulb elicits changes in the area under the ROC for the odorant-induced change in power evaluated as an event-related LFP locked to lick onset

Studies in medial prefrontal cortex have shown that the LFP in the theta (6–12 Hz) bandwidth is phase-locked to licks, even when the animal performs dry licks in a rewarded task (Amarante et al., 2017). For the olfactory system this could be interesting, particularly considering that orofacial control of licks and sniffing are related through control of brainstem central pattern generators by cortex, basal ganglia, and cerebellum (Moore et al., 2014) and that sniffing is tightly linked to the theta LFP in the OB (Grosmaitre et al., 2007; Rosero and Aylwin, 2011; Gschwend et al., 2012; Khan et al., 2012). We proceeded to analyze the LFP as an event-related potential in phase with the dry licks that the mouse is performing during the application of the odorant (Figure 1E). We refer to this LFP as the lick-related LFP (LR-LFP).

**Figure 7:**
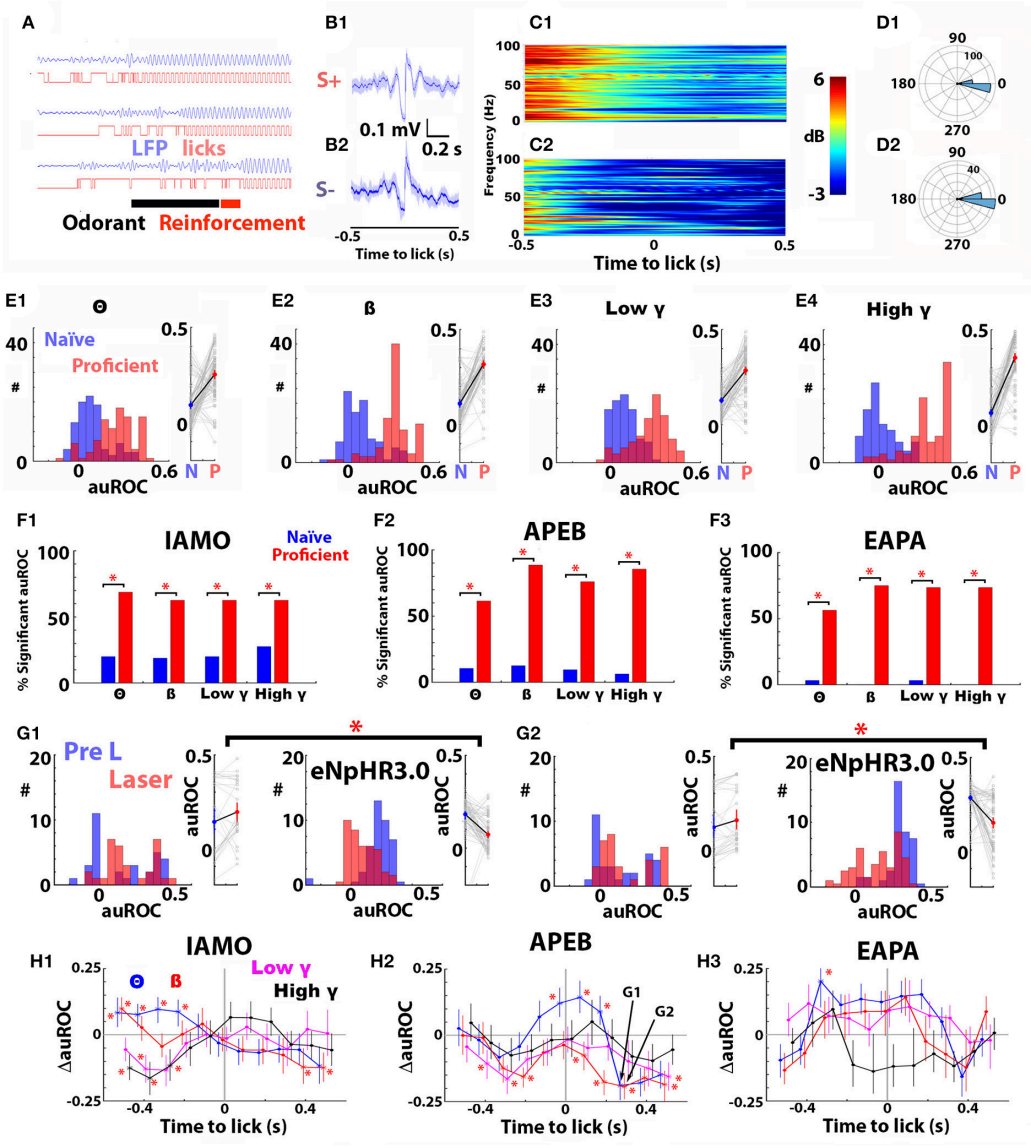
Optogenetic silencing of norepinephrine axons in the olfactory bulb elicits statistically significant changes in the auROC for the odorant-elicited change in power of the lick-related LFP (Δ power LR-LFP). **(A)** Examples of traces showing phase locking of the theta LFP filtered at 6–12 Hz with licks for three trials when the animal was proficient in differentiating odorants for the APEB odorant pair in the go-no go task. Licks were detected as an increase in voltage elicited when the tongue touched the lick spout. The black bar shows the duration of odorant application (2.5s). **(B)** Mean LFP and 95% confidence intervals, computed by bootstrapping, recorded when the LFP was triggered by the onset of the lick, for all licks occurring for 2 s after odorant application for 30 trials for the mouse whose raw traces are shown in **(A)** (**B1**, S+, **B2** S–). The lick-locked LFP is shown for the time interval from −0.5 to 0.5 s centered at lick onset (lick-related LFP, LR-LFP). **(C)** Spectrogram for the odorant-induced change in LR-LFP power for S+ **(C1)** and S– **(C2)** for these 30 trials. **(D)** Theta LFP phase of the lick onset for S+ **(D1)** and S– **(D2)** for these 30 trials. **(E1–E4)** The auROC for Δ power LR-LFP computed for 30 S+ and S– trials increases for all bandwidths between the first 30 trials of the first go-no go training session (naïve, blue) and the last 30 trials of the last training session (proficient, red) for animals learning to discriminate the APEB odorant pair. **(A1)** theta, **(A2)** beta, **(A3)** low gamma, **(A4)** high gamma. For each panel a histogram of auROC values is shown on the left, and a scatter plot is shown on the right. For all bandwidths the *p*-value for a permuted N-way ANOVA testing for the difference in auROC between naïve and proficient was <0.0001, pFDR = 0.05 (9 mice, 16 electrodes each). **(F1–F3)** The percent of significant auROCs for Δ power LR-LFP computed for 30 S+ and S–trials increases for all bandwidths between the first 30 trials of the first go-no go training session (naïve, blue) and the last 30 trials of the last training session (proficient, red) for animals learning to discriminate the IAMO **(F1)**, APEB **(F2)**, and EAPA **(F3)** odorant pairs (percent significant auROC is shown for all bandwidths). The number of significant auROCs differed between naïve and proficient trials for all odorant pairs and all bandwidths when tested with a Chi Squared test (*p* < pFDR = 0.05, number of mice 7 for IAMO, 9 for APEB, and 9 for EAPA, 16 electrodes per mouse). **(G)** Examples of the effect of optogenetic silencing of the noradrenergic fibers in the OB on the Δ power LR-LFP auROCs calculated with Hits and CRs for the last 30 trials in the session where the animal was proficient in differentiating odorants for the APEB odorant pair (Pre L, blue) and the first 30 trials in a subsequent session where light was applied for 3.5 s starting when the animal entered the port (Laser, red). Shown are the histograms (left) and scatter plots (right) for Δ power LR-LFP auROCs for theta **(G1)** and beta **(G2)** bandwidths before (Pre L, blue) and during (Laser, red) laser stimulation for the APEB odorant pair. Data are shown for DBH-Cre mice (**C1**,**C2**, left, *n* = 4, 16 LFP electrodes each) and DBH-Cre eNpHR3.0 mice (**C1,C2**, right, *n* = 6, 16 LFP electrodes each). The auROC in panels **(C1,C2)** was calculated using LR-LFP Δ power recorded in Hit and CR trials 0.3 s after the onset of the lick. The interaction term of an N-way ANOVA with mouse genotype and laser stimulation as the two factors was significant for the Hit/CR auROC for both **(C1,C2)** (*p* ≤ 0.0001, <pFDR = 0.022). H. Difference between genotypes of the change in auROC elicited by optogenetic silencing of local noradrenergic fibers (ΔauROC = change in auROC for DBH-Cre eNpHR3.0 mice—change in auROC for DBH-Cre mice). Shown are the mean and the estimate of the 95% confidence intervals for the ΔauROC for all bandwidths for IAMO **(H1)**, APEB **(H2)**, and EAPA **(H3)**. Data for the different bandwidths are shown in different colors: theta (blue), beta (red), low gamma (purple), and high gamma (black). The LR-LFP Hit/CR auROC was computed as shown for examples in panel **(G)**. In H2 arrows points to ΔauROC data points corresponding to the data shown in panels **(G1)** (theta, APEB) and **(G2)** (beta, APEB). Asterisks denote ΔauROCs found to be significant for the interaction term of an N-way ANOVA with mouse genotype and laser stimulation as the two factors (*p* ≤ pFDR, pFDR = 0.015 for IAMO, 0.022 for APEB and 0.001 for EAPA, the number of DBH-Cre mice was 5 for IAMO, 4 for APEB, and 8 for EAPA and the number of DBH-Cre eNpHR3.0 mice was 4 for IAMO, 6 for APEB, and 4 for EAPA, 16 electrodes per mouse).

Figure 7A shows examples of theta LFP and lick traces for three trials recorded when a mouse was proficient in discrimination of the odorants for the APEB odorant pair and Figures 7B–D present the analysis of the LR-LFP computed for all licks occurring in for 2 s after delivery of the odorant during the last 30 trials of a session when the mouse was proficient for the APEB odorant pair. Lick onset was found to be locked to theta LFP phase for both S+ and S– odorants (Figure 7D). When examined visually, the mean LR- LFP appeared different between S+ and S– (Figure 7B), and a spectrogram analysis of the odorant-induced change in the power of the LR-LFP appeared to show differences between S+ and S– trials in Δ power for the LR-LFP (Figure 7C).

We proceeded to analyze the changes in LR-LFP Δ power when the mice became proficient by calculating the auROC for LR-LFP Δ power using the same analysis strategy performed for LFP Δ power in Figure 3. Figure 7E shows that for all bandwidths the LR-LFP Δ power auROC computed in the first 30 trials of the first session, when the mouse is naïve to APEB odorant value (naïve, blue) is smaller than the LR-LFP Δ power auROC computed for the last 30 trials of the session when the animal is proficient in the discrimination of these odorants (proficient, red). The difference in LR-LFP Δ power auROCs between naïve (blue) and proficient (red) is statistically significant for all bandwidths when tested using a non-parametric permutation based ANOVA (Delorme and Makeig, 2004) for all odorant pairs (*p* < 0.001, pFDR = 0.05, the number of mice was 7 for IAMO, 9 for APEB, and 9 for EAPA, IAMO, and EAPA data are not shown). In addition, the percent significant LR-LFP Δ power auROCs increased significantly between the naïve and proficient states (Figure 7F). Thus, as we found for LFP Δ power, learning elicits a significant increase in the ability to discriminate between odorants using the LR-LFP Δ power values in the S+ and S– trials.

We then proceeded to determine whether optogenetic silencing of noradrenergic fibers elicited a change in LR-LFP Δ power auROC in DBH-Cre eNpHR3.0 mice expressing eNpHR3.0 in LC. The auROC analysis for LR-LFP Δ power was performed in the same manner as it had been performed for the auROC Δ power LFP analysis in Figure 6 including a comparison with DBH-Cre mice that do not express eNpHR3.0. Thus, we compared the auROCs for LR-LFP Δ power for the last 20 trials of the session when the animal had become proficient at differentiating between odorants to the auROC calculated for the first 20 trials of the subsequent session where the laser was turned on for 3.5 s when the animal entered the port. Importantly, this analysis was performed using Hits and CRs for the same sessions and same mice used for the auROC Δ power LFP analysis in Figure 6 where this analysis did not yield an interaction of genotype and laser application.

Figure 7G shows histograms and scatter plots of the effect of local optogenetic silencing on the LR-LFP Δ power auROC calculated 0.3 s after the lick for Hits and CRs for the theta bandwidth for control DBH-Cre (Figure 7G1, left) and DBH-Cre eNpHR3.0 mice (Figure 7G1, right) discriminating the APEB odorant pair. Figure 7G2 shows the corresponding auROC histograms and scatter plots for the beta bandwidth with the same odorant pair. Optogenetic silencing of noradrenergic fibers elicited a substantial decrease in the LR-LFP Δ power auROC for the mice expressing eNpHR3.0 (Figures 7G1,G2, right). When tested with an N-way ANOVA for the interaction between light activation and genotype, there was a significant differential effect for both theta and beta (*p* < 0.0001 < pFDR = 0.022, 4 DBH-Cre mice and 6 DBH-Cre eNpHR3.0 mice, 16 electrodes each). In order to display the effect of opsin silencing on the LR-LFP Δ power auROC at different times with respect to the onset of the lick for the different bandwidths we calculated the difference in the light-induced change in auROC between the two genotypes (ΔauROC = light-induced change in auROC for DBH-Cre eNpHR3.0 mice—change in auROC for DBH-Cre mice). Figure 7H shows that significant ΔauROC changes elicited by optogenetic silencing occurred for all odorant pairs tested. Asterisks in Figure 7H denote ΔauROCs found to be significant for the interaction term of an N-way ANOVA with mouse genotype and laser stimulation as the two factors (*p* ≤ pFDR, pFDR= 0.015 for IAMO, 0.022 for APEB and 0.001 for EAPA, the number of DBH-Cre mice was 5 for IAMO, 4 for APEB and 8 for EAPA and the number of DBH-Cre eNpHR3.0 mice was 4 for IAMO, 6 for APEB and 4 for EAPA, 16 electrodes per mouse). Significant positive and negative changes in ΔauROCs were found at different times with respect to the onset of the lick. This analysis indicates that optogenetic silencing of local noradrenergic fibers causes a significant change in the ability to differentiate between odorants using the odorant-induced power of the LFP.

## Discussion

The olfactory bulb is the first brain area where the input from olfactory sensory neurons is processed. Thus, it is analogous to the retina in the visual system. However, the olfactory bulb is strikingly different to the retina because it receives massive centrifugal feedback from axons in the piriform (olfactory) cortex, and from modulatory neurons such as cholinergic neurons in the horizontal limb of the diagonal band of Broca and the noradrenergic fibers from the LC (Gire et al., 2013) #1799. This striking difference raises the question whether signal processing in the olfactory bulb is exclusively dedicated to processing of sensory features such as quality and intensity, or whether the olfactory bulb also receives information on higher order qualities of the sensory stimulus such as odorant value, and whether the modulatory input alters such higher order signal processing in the bulb.

In this study, we determined whether local optogenetic silencing of adrenergic axons in the OB alters the capability to classify, using differences in odorant-elicited changes in oscillatory power (Δ power), the odorant as rewarded vs. unrewarded in the go-no go odorant discrimination task. We found development of a difference in the odorant-induced Δ power between rewarded and unrewarded odorants as the animal learned to differentiate between odorants. A ROC analysis indicated that, for the majority of the LFPs recorded in the study, Δ power did not carry information on the difference between the odorants at the start of the task. In contrast, Δ power could be used to classify the odorant when the animal became proficient. In addition, ROC analysis indicated that Δ power could be used to classify the trials for the unrewarded odorant between false alarms and correct rejections and the polarity of odorant-induced Δ power reversed when the rewarded odorant was switched indicating that this measure caries information on odorant value, as opposed to odorant identity. Finally, local optogenetic silencing of adrenergic axons in the OB resulted in changes in the area under the ROC when the LFP was aligned to the onset of the lick indicating that noradrenaline modulates local circuit processing of oscillations carrying information on odorant value.

Gamma oscillations reflect local synchronized neural activity while slower oscillations arise because of neural communication between different brain regions (Buzsáki, 2010; Kay, 2014; Martin and Ravel, 2014). Previous studies in rodents undergoing learning in a go-no go odorant discrimination task have yielded discrepant results on which bandwidths were involved in development of a differential odorant-induced change in oscillatory power as the animals learn to differentiate between odorants. Martin et al. (2004) showed development of a differential response in beta oscillations as rats learned to discriminate between two odorants in a go-no go task (Martin et al., 2004). They found a larger increase in beta power for S+ compared to S– that developed during learning. This differential beta response differed between electrode locations. They concluded that the neural activity reflected by the beta oscillations was involved in learning. In contrast, studies by Beshel et al. (2007) showed development of a differential gamma response in rats undergoing a two-alternative forced choice (2AFC) task. They raised the question whether the difference with the study by Martin and co-workers was due to the difference in the behavioral tasks (go-no go vs. 2AFC). Finally, studies from our laboratory recorded oscillations in the OB of mice undergoing the go-no go odorant discrimination task (Li et al., 2015a). We found development of an increase in power for theta oscillations, but not for gamma.

The recordings shown in this study show development of a broadband increase in oscillatory power as the mice learn to differentiate two odorants in the go-no go odorant discrimination task. Why are there discrepancies between the different studies? The differences could be due to the use of different species (mice vs. rats) or the location of electrodes (the present study placed the electrodes in a dorsal location while our earlier study placed them ventromedially). However, it is likely that the differences are due to differences in behavioral demands in the different tasks. Indeed, Frederick and co-workers examined oscillations in rats undergoing go-no go and/or 2AFC tasks (Frederick et al., 2016). They concluded that participation of beta and gamma oscillations depends on cognitive and sensory demands of the particular tasks. In our opinion, understanding of the differential involvement of neural circuits generating oscillations in particular bandwidths is an open question that needs to be addressed in future experiments that use novel computational and approaches to understand the neural basis of these oscillations paying particular attention to differences in behavioral demands.

Here we find that local optogenetic silencing of adrenergic axons in the olfactory bulb alters the auROC for oscillations in all bandwidths.

This is consistent with previous findings that local infusion of adrenergics antagonists in the olfactory bulb alter synchronized firing of mitral/tufted cells divergent to the reinforced and unreinforced odorants in the go-no go task (Doucette et al., 2011). In addition, studies of circuit dynamics in olfactory bulb slices indicate that adrenergic receptor activation leads to long term enhancement of low gamma frequency oscillations in the olfactory bulb (Pandipati et al., 2010), however this effect was not observed in animals older than P14 (Pandipati and Schoppa, 2012). Finally, computational modeling of the effect of adrenergic modulation on circuit activity and oscillations in the olfactory bulb indicates that adrenergic modulation of oscillatory activity may increase the signal to noise ratio of the circuit (de Almeida et al., 2015; Li et al., 2015b).

What is the behavioral significance of NA modulation of sensory processing in the OB? The experiment used in this study was not designed to determine whether local optogenetic silencing of noradrenergic fibers alters behavioral performance because light activation was unilateral and was limited to a small area in the olfactory bulb due to the tight focus of the 0.22 NA 105 μm diameter optical fiber and to quasi-exponential reduction of light intensity as a function of tissue depth because of light scatter (Stujenske et al., 2015). However, we did find a small statistically significant decrease in performance in discrimination for the IAMO odorant pair (Figure 6E2). This suggests that noradrenergic modulation plays a role in responding to the rewarded odorant in the go-no go odorant discrimination task. This is consistent with previous studies from our group that showed that local infusion of adrenergic inhibitors in the OB abolished odorant discrimination in the go-no go task for a subset of the odorants tested (Doucette et al., 2007). Thus, likely the biased innervation of the OB by LC-NA axons (Schwarz et al., 2015) plays an important role in mediating a response to the rewarded odorant. However, future experiments are required to determine whether this is the case.

Why is information on odorant value processed in the olfactory bulb? The answer to this question is not evident. However, we speculate that since the olfactory input has a large number of degrees of freedom because of the large number of olfactory receptors this information is used for local query of the incoming signal. Whether this is correct requires further studies.

In summary, we find that broadband odorant-induced changes in LFP power can be used to classify the odorants as rewarded or unrewarded. Broadband spectral changes in cortical surface recordings have been found to be predictive of visual stimuli with high accuracy (Miller et al., 2016). However, the broadband odorant-induced changes in LFP power do not represent odorant identity, they reflect odorant value. In addition, we find that local optogenetic silencing of adrenergic axons in the olfactory bulb decreases the ability to classifying the odorants using odorant-induced changes in lick-aligned LFP power in the theta, beta and gamma frequencies. These results suggest that noradrenergic modulation of local circuit processing in the olfactory bulb play a role, not only in the identification of odorant quality, but also in evaluation of odorant value, or that the information on odorant value is used to query the high dimensional space of odorant input.

## Author contributions

DR-G and DR: designed the experiments; DR-G: performed the awake behaving recording and optogenetic modulation experiments; MM: performed the histology; DR-G and DR: performed data analysis. All authors wrote the paper.

### Conflict of interest statement

The authors declare that the research was conducted in the absence of any commercial or financial relationships that could be construed as a potential conflict of interest.
